# Analysis of Vibration, Deflection Angle and Surface Roughness in Water-Jet Cutting of AZ91D Magnesium Alloy and Simulation of Selected Surface Roughness Parameters Using ANN

**DOI:** 10.3390/ma16093384

**Published:** 2023-04-26

**Authors:** Katarzyna Biruk-Urban, Ireneusz Zagórski, Monika Kulisz, Michał Leleń

**Affiliations:** 1 Department of Production Engineering, Mechanical Engineering Faculty, Lublin University of Technology, 20-618 Lublin, Poland; k.biruk-urban@pollub.pl (K.B.-U.); m.lelen@pollub.pl (M.L.); 2 Department of Enterprise Organisation, Faculty of Management, Lublin University of Technology, 20-618 Lublin, Poland; m.kulisz@pollub.pl

**Keywords:** water-jet cutting, magnesium alloys, vibration, roughness, simulations, artificial neural networks ANN

## Abstract

The use of magnesium alloys in various industries and commerce is increasing due to their properties such as high strength and casting properties, high vibration damping capability, good shielding of electromagnetic radiation and high machinability. Conventional machining methods can, however, pose a risk of ignition. AWJM is a safe alternative to conventional machining, but the deflection and vibration of the water jet can affect surface quality. Therefore, the aim of this study was to investigate the effects of selected AWJM parameters on the surface quality and vibration of machined magnesium alloys. Jet deflection angle, surface roughness parameters and vibration during AWJM were investigated. The findings showed that higher skewness occurred at a lower abrasive flow rate, while higher average values of the Sku roughness parameter were obtained at m_a_ = 8 g/s in the range of 60–140 mm/min. It was also observed that higher vibration values occurred at m_a_ = 8 g/s. The input parameters for creating an artificial neural network (ANN) model used in this study were the cutting speed v_f_ and the mass flow rate m_a_. The results of this study provided valuable insights into ways of ensuring a safe and efficient machining environment for magnesium alloys. The use of ANN modeling for predicting the vibration and surface roughness of AZ91D magnesium alloy after water-jet cutting could be an effective tool for optimizing AWJM parameters.

## 1. Introduction

Among the different structural materials, magnesium alloys offer both energy efficiency and environmental benefits, which makes them one of the most versatile choices. Magnesium is characterized by high strength and casting properties, high vibration damping capacity, good electromagnetic radiation shielding [1] and high machinability, which means that even very complicated parts can easily be machined with high dimensional accuracy [2]. Various industries and commercial sectors, such as automotive, aviation, defense, biomedical, sporting equipment and consumer electronics, can benefit from magnesium-based materials (alloys and composites) [3]. Magnesium is characterized by a hexagonal crystal lattice, which results in its poor ductility, so most magnesium alloy parts are produced by casting processes. These parts often require machining [4]. Machining processes for magnesium alloys include turning [5], drilling [6], threading [7], milling [8] and water-jet cutting [9].

The conventional machining of magnesium-based materials poses ignition problems. In turning and milling processes where the machining temperature reaches about 450 °C, the risk of ignition increases. To ensure the safest possible machining environment, abrasive water jet machining (AWJM) can be used for magnesium alloys [10]. The surface after AWJM is very smooth, so finishing is not required. In addition, the AWJM process has no effect on the condition of workpiece material (apart from plastic deformation), as there is no heat-affected zone (contrary to laser and plasma cutting), which maintains the integrity of the alloy structure [11]. The input parameters of AWJM, such as hydraulic parameters (orifice diameter, water pressure), abrasive parameters (abrasive flow rate AFL, abrasive material and size) and cutting parameters (traverse speed TS, stand-off distance SOD, angle of attack) have been determined in numerous studies [9,12,13,14,15] to be the factors defining surface quality after AWJM. Therefore, this study investigates the impact of specific AWJM parameters on the surface quality and vibration of magnesium alloys to ensure a safe machining environment.

**Figure 1:**
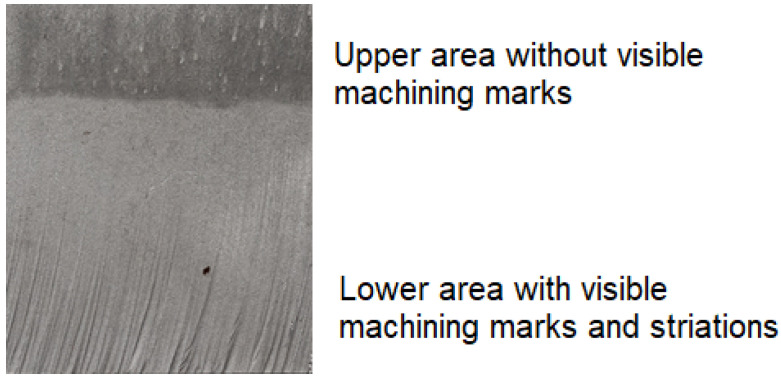
Surface of an AZ91D specimen after AWJM.

Water jet deflection occurs at a certain thickness of material treated by AWJM. This results from the fact that most water jet energy removes the upper layer of the machined sample, and the remaining energy is unable to cut the material as effectively as at the beginning of the process (on the upper side of the sample) [16]. Figure 1 shows the situation when the depth of water jet impact in the material is increased, and the quality of the machined surface becomes worse. There are visible machining marks and striations on the deeper surface, which results from an uneven distribution of kinetic energy of the abrasive material.

One of the indicators analyzed in this paper is the deflection angle of the jet. This aspect has not been investigated in many previous works, as most studies focus on the kerf angle depending on the kerf depth and width [17]. Khan and Gupta [18] considered the cutting angle for low alloy steel EN24 samples of different thicknesses. The results showed that the angle increased with increasing sample thickness. Alsoufi et al. [19] studied the influence of different technological parameters of water-jet machining on the water-jet angle. It was found that the dimension of the area of visible machining marks and their angle increased with increasing the cutting traverse rate and that there was a direct relation between the technological parameters and the jet deflection angle.

Another indicator analyzed in this paper is vibration occurring during AWJM. In the AWJM process, the abrasive water-jet particles hit the machined surface of the workpiece at high speed, which generates vibration in the workpiece and, additionally, acoustic signals [20]. Peržel et al. [21] investigated the vibration generated by abrasive water-jet (AWJ) cutting of stainless steel with different abrasive mass flow rates of 250 and 400 g min^−1^ and a constant traverse speed. Based on the measured amplitudes and frequency spectrum, a relation was established between the input factors of AWJM and the vibration and acoustic emissions. Tyč et al. [22] analyzed the vibration signals generated in the AWJ cutting of hard-to-machine materials (RSt 37-2 steel) with five different sample thicknesses. In the study, they used three accelerometers mounted on a special stand for monitoring vibration. They found that the root mean square (RMS) value of a vibration signal was related to traverse speed. The RMS increased with the increasing traverse speed and depended on the direction of vibration measurement by the accelerometer. Krenický and Rimár [23] studied vibrations to analyze the operational parameters of AWJ cutting. They divided vibration into exogenous (workpiece vibrations caused by the jet interacting with the workpiece, equipment and water in the tank) and endogenous (vibrations of the jet coming from the cutting head). Vibrations generated during the tests were reduced by using nozzle stabilization and isolation from the positioning system vibration, and for the exogenous vibration, by the application of a specially designed fixing device for workpiece mounting. A study conducted by Karminis-Obratański et al. [24] aimed to determine whether vibration measures could be used for AWJ effectiveness monitoring. The study found that even though there was no direct correlation between process effectiveness and vibration amplitude, a tendency was observed for average vibration amplitude to increase with depth and width. This phenomenon was justified by the occurrence of higher kinetic energy and momentum of the abrasive water stream.

The quality of a machined surface can be measured based on surface roughness, waviness and surface defects. Surface roughness is one of the machining process efficiency evaluation indicators and hence is the most widely used [25]. Roughness measurements may relate to 2D surface profile parameters and stereometric characteristics of surface roughness (3D) [26]. The parameters Ra (arithmetic mean profile deviation) and Rz (cusp height of the profile) are most often analyzed; however, for a full description of the geometrical condition of the surface, other roughness parameters should also be taken into account. The factors directly influencing surface roughness are technological parameters such as traverse speed, material grade and thickness, abrasive flow rate and abrasive size. Löschner et al. [27] investigated the influence of cutting speed on surface roughness, its quality and the presence of machining marks. They found that cutting speed and distance from the upper cut surface edge had the greatest impact on surface roughness. Skoczylas et al. [28] also found that cutting speed had the greatest impact on surface roughness, regardless of the type of material. The surface roughness in the entry and exit zones was examined, and it was found that the entry zone was characterized by lower roughness (Ra parameter was analyzed). However, when the cutting process was conducted with low cutting speeds, the differences in surface roughness in the entry and exit zones were very small; nevertheless, the differences would increase with increasing speed. Deaconescu et al. [29] conducted a study aimed at optimizing the AWJ process for stainless steel to obtain minimum roughness parameters. Their study showed that increased water-jet pressure led to reduced surface roughness. To ensure good surface quality, they recommended using low traverse speed and stand-off distance values.

Artificial intelligence methods, including artificial neural networks, have been increasingly used in research. Artificial neural networks are widely used in various predictive applications. The ability of ANN models to predict non-linear systems and the ease of their implementation contributed to their increased use for solving research problems connected with aspects, such as the prediction of, e.g., hot flow stress [30] or high-temperature deformation of steel [31], chemical composition modeling [32], industrial electrical tomography [33], electrical impedance tomography [34]. Modeling has also been applied in studies on abrasive water-jet machining. Ganovska et al. [35] analyzed a selection of roughness parameters (Ra, Rq and Rz), technological parameters (traverse speed, abrasive mass flow rate) and vibration in the AWJC process for stainless steel. Furthermore, the equations for predicting surface roughness parameters were derived. It was found that the surface topography depended on the traverse speed of the cutting head. Ficko [36] also analyzed the impact of machining stainless steel by AWJ with selected technological parameters (traverse speed, depth of cut and abrasive mass flow rate) on the surface roughness (Ra) of this material. The obtained test results were used for creating a predictive model of the Ra parameter with the use of an artificial neural network (ANN). It was found that the proposed model could be applied to optimizing AWJ process parameters. Zagórski et al. [11] investigated the surface condition of alloy AZ91D by predicting roughness parameters Ra, Rz and RSm after AWJM conducted with variable technological parameters, notably a cutting speed v_f_ and an abrasive flow rate m_a_.

A review of the literature showed that the majority of previous studies on water-jet cutting address the problem of surface quality based on the evaluation of 2D roughness parameters. However, it is also worth considering 3D roughness parameters for a more accurate assessment of surface quality. Rku, Rsk, Sku and Ssk are important from the point of view of operating parameters, as they affect the tribological properties of a surface, such as friction, wear and wear resistance. In fact, 3D roughness parameters can better reflect the actual properties of the surface and their impact on downstream processes such as adhesion and corrosion. The quality of the surface after water jet cutting depends on many factors, including the applied technological parameters of the process. Therefore, it is important that these parameters should be selected correctly to obtain the desired surface quality and to ensure a safe and efficient machining environment for magnesium alloys.

## 2. Materials and Methods

**Figure 2:**
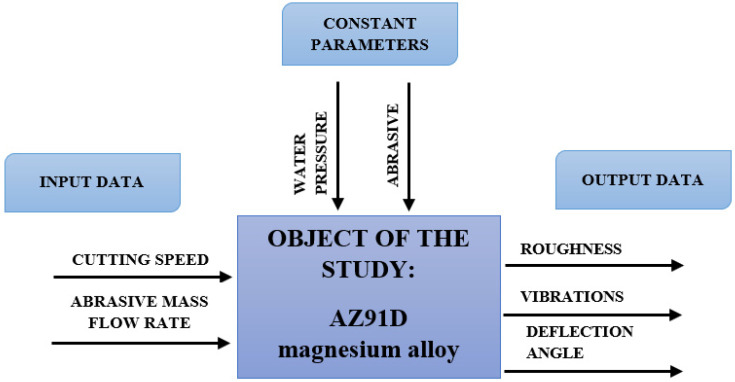
Scheme of the research plan.

The main aim of this study was to determine the influence of variable technological parameters of AWJM, such as cutting speed and abrasive mass flow rate, on the vibration and surface quality of magnesium alloy AZ91D. Results of the study will make it possible to define optimum technological parameters of AWJM, ensuring high surface quality and machining safety. A research plan is presented in Figure 2.

### 2.1. Materials and Machining Method

Alloy AZ91D, which is the most widely used magnesium cast alloy, was the subject of this research. AZ91D bars were machined on a WaterJet Combo abrasive water jet cutter (Legnica, Poland) equipped with a CNC ECS 872 controller. This machine allows cutting various types of materials with an abrasive water jet and a plasma jet. As a result of the AWJM process, the following sample dimensions were obtained: 100 × 56 × 20 mm, where the height of the sample was equal to the cutting height and was 56 mm. The abrasive medium was GARNET 80 (almandine garnet). The diameter of the nozzle was 0.7 mm, the distance between the nozzle and the material was 3 mm, the length of the nozzle was 100 mm, the jet impact angle was 90° and the water pressure was 350 MPa.

Variable technological parameters of the AWJM process included a cutting speed v_f_ of 5–180 mm/min and an abrasive flow rate m_a_ of 4 and 8 g/s. The values of these parameters were determined experimentally based on previous studies and literature reviews, and they are listed in Table 1.

### 2.2. Measurement Methods

#### 2.2.1. Vibration

**Figure 3:**
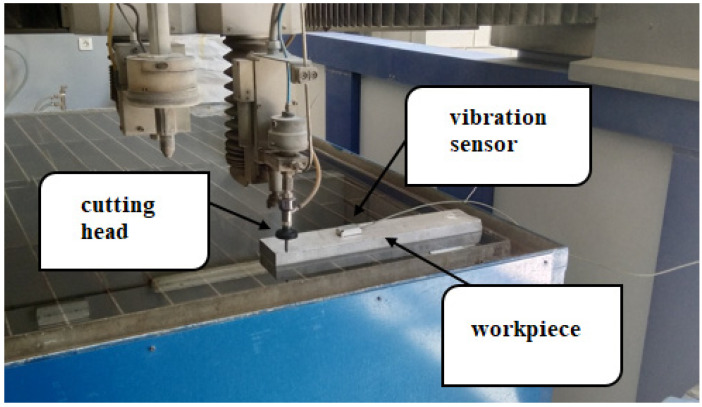
Test stand for vibration measurement.

During the AWJM of AZ91D magnesium alloy samples, vibration was measured on a test stand (Figure 3) for each of the variable parameters. A Sequoia sensor used in the tests was located in the central part of the sample, at a distance of 100 mm from the edge of the sample. The distance of the sensor from the cut surface was maintained constant for every tested parameter. The vibration measurements were made for all technological parameters applied in the study.

#### 2.2.2. Surface Roughness

The AWJM process was followed by measurement of 2D and 3D surface roughness. The measurements of the 2D surface roughness of the samples after the AWJM process conducted with variable technological parameters were made on the Hommel T1000 (Jena, Germany) contact profilometer in five repetitions at two measuring points. The first measurement point was located in the middle of the sample height, and the other in the area of water jet entry. The roughness measurements were made with the following technological parameters: ISO 11562 filter (M1), sampling length Lc = 0.8 mm, measuring length Lt = 4.8 mm, traverse feed velocity v after AWJM = 0.5 mm/s. The sample surface scan area was 1.6 × 1.6 mm with 100 scan steps. In the tests, the following 2D surface roughness parameters were measured: Rku and Rsk. Rku and Rsk are roughness parameters describing surface properties. They are widely used to determine surface quality and its degree of roughness. Rku describes roughness along the main direction of motion, while Rsk describes roughness perpendicular to the main direction of motion. These parameters are important because they affect tribological properties of a surface, such as friction as well as wear and tear resistance. In addition, Rku and Rsk are often used to determine manufacturing quality and surface quality control. The 3D surface roughness of the samples after AWJM was conducted with variable parameters was measured with the Hommel T8000 RC120–400 device. The measurements included the determination of Sku and Ssk and were carried out perpendicular to the machining marks. These parameters also affect tribological properties of surfaces, such as friction as well as resistance to wear and tear. In addition, Sku and Ssk are often used to compare the surface quality of different materials and to determine their suitability for specific applications.

#### 2.2.3. Deflection Angle of the Jet Vibration

**Figure 4:**
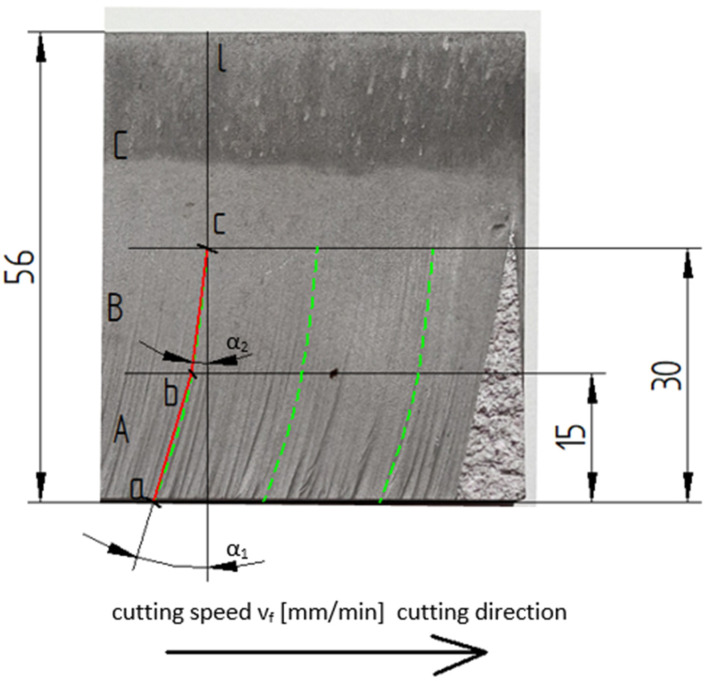
Method of measuring deflection angle.

An analysis of jet deflection angle was performed on the Keyence VHX-5000 (Osaka, Japan) microscope, with a magnification of ×100. Figure 4 presents the surface of an AZ91D sample cut with a cutting speed v_f_ of 80 mm/min and an abrasive flow rate of 8 g/s. One can notice visible marks of the machining process and of the employed deflection angle measurement method. The surface of the machine sample was divided into three areas marked with letters A, B, C. The first area was marked with letter A. The surface of this area extends from the bottom of the sample edge to a height of 15 mm. In this area, the deflection angle of the jet was marked as α_1_. The α_1_ angle is between section a–b and straight line l, perpendicular to the cutting direction. In area B, the α_2_ angle was marked between section b–c and straight line l, perpendicular to the cutting direction. The dotted line marks the course of the jet of the cut material. Point a is the starting point of measurement. Point b denotes the intersection of a deflection curve with a straight line at a height of 15 mm. Point c is the point of intersection of the jet deflection curve’s end with a straight line at a height of 30 mm. In area C, however, the deflection angle of the jet was not measured due to a lack of visible characteristic marks. The deflection angle of the jet was measured in two areas: A and B.

### 2.3. Artificial Neural Network

**Figure 5:**
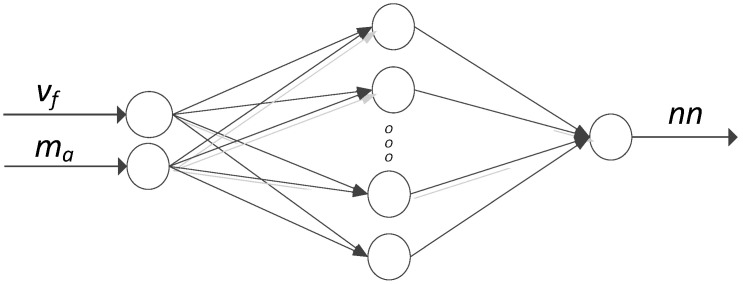
Schematic structure of the modeled neutral network, where nn stands for a correctly modeled parameter.

To predict the non-linear AWJM process for AZ91D, models of selected roughness parameters 2D (Rku) and 3D (Sku) were created. The predictive models were constructed with artificial neural networks using Matlab R2021b (The MathWorks, Inc., Natick, MA, USA). Given that the aim of modeling was to obtain the simplest network structure with one hidden layer, a shallow neural network was used. The input layer consisted of two neurons (cutting speed v_f_ and abrasive flow rate m_a_), while the output layer consisted of one neuron (respective roughness parameters). A schematic structure of the neural network is presented in Figure 5, where appropriate roughness parameters are obtained at the output of the model.

The Levenberg–Marquardt algorithm was used for network training. The number of neurons in the hidden layer (2–10) was selected experimentally, and the maximum number of training epochs was 1000. The hyperbolic tangent sigmoid transfer function was used as an activation function. The training data accounted for 75% of the measurement results and 25% of the validation data. Due to the small number of data sets, the test data [37] were abandoned. The most important indicators of network selection correctness were a regression value R (correlation coefficient), mean squared error (MSE) and root mean square error (RMSE).

The regression value R was calculated in accordance with the formula:

$$R\left(y^{\prime },y^{*}\right)=\frac{\mathrm{cov}\left(y^{\prime },y^{*}\right)}{\sigma_{y^{\prime }}\sigma_{y^{*}}}\;R\in <0,1>$$

where:

σ_y_′—standard deviation of the roughness parameters value obtained experimentally;

σ_y_*—standard deviation of the roughness parameters value obtained as a result of prediction.

The MSE value and root mean square error (RMSE) were calculated in accordance with the formulae:

$$\mathrm{MSE}=\frac{1}{n}\overset{n}{\underset{n=1}{\sum }}{\left({\hat{y}}_{i}-y_{i}\right)}^{2}$$

$$\mathrm{RMSE}=\sqrt{\frac{1}{n}\overset{n}{\underset{n=1}{\sum }}{\left({\hat{y}}_{i}-y_{i}\right)}^{2}}$$

where:

$y_{i}$—value of a specified roughness parameter for the i-th observation obtained experimentally;

${\hat{y}}_{i}$—value of a specified roughness parameter for the i-th observation obtained as a result of prediction.

## 3. Results and Discussion

### 3.1. Vibration

**Figure 6:**
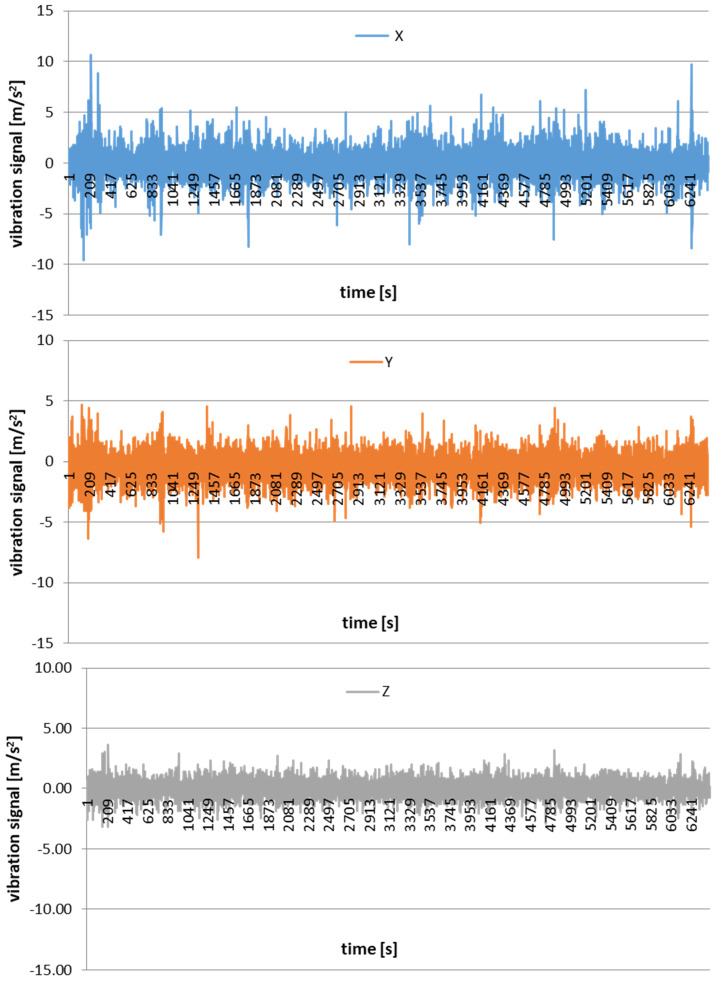
Examples of graphs showing vibration signal in the *X*-, *Y*- and *Z*-axis.

Figure 6 shows examples of time courses of vibration acceleration in the AWJ cutting of AZ91D magnesium alloy. The results are given for the following machining conditions: v_f_ = 5 mm/min, m_a_ = 8 g/s. The vibration versus time results are shown separately for every component analyzed in the *X*-, *Y*- and *Z*-axis direction.

**Figure 7:**
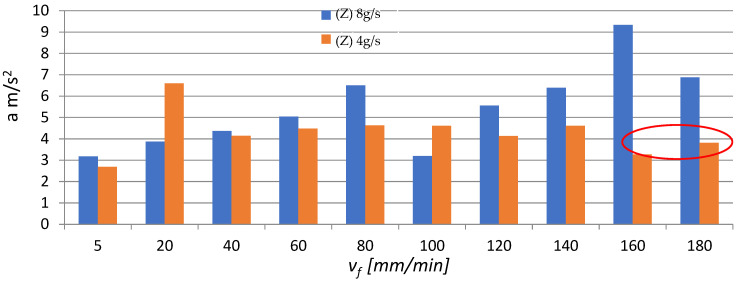
Influence of different v_f_ values on the maximum vibration acceleration for two abrasive flow rates: m_a_ = 8 g/s and m_a_ = 4 g/s.

**Figure 8:**
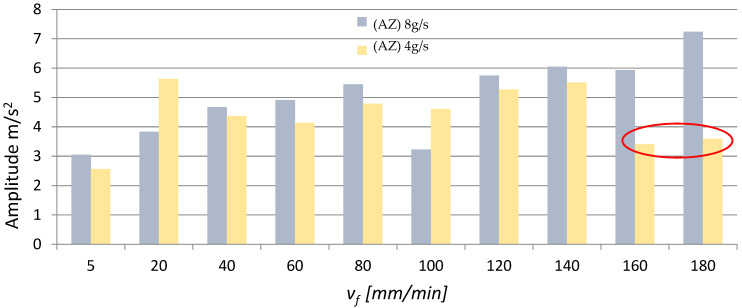
Influence of different v_f_ values on the amplitude of vibration acceleration for two abrasive flow rates: m_a_ = 8 g/s and m_a_ = 4 g/s.

**Figure 9:**
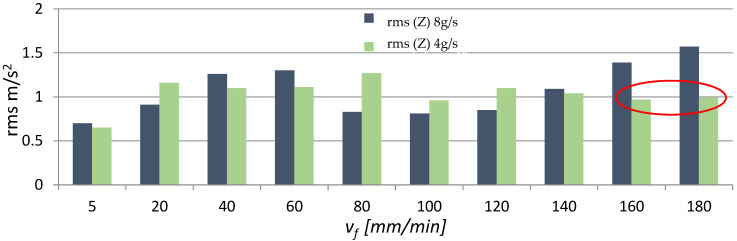
Influence of different v_f_ values on rms values of vibration acceleration for two abrasive flow rates: m_a_ = 8 g/s and m_a_ = 4 g/s.

In addition, time signals were used to investigate changes in the amplitude, maximum value and effective value (rms) of vibration acceleration. Figure 7, Figure 8 and Figure 9 present the effect of different values of v_f_ on the maximum value, amplitude and rms of vibration acceleration.

An analysis of the data in Figure 7, Figure 8 and Figure 9 reveals the presence of a characteristic area where the vibration (its maximum value, amplitude and root mean square) rapidly decreases in the speed range v_f_ = 160–180 mm/min (the area is marked in red circles in Figure 7, Figure 8 and Figure 9). This is due to the fact that the workpiece did not undergo AWJ cutting with the applied machining parameters. Furthermore, magnesium alloys have good damping properties, which could additionally affect the value and level of vibration. Regarding the remaining v_f_ range, it can roughly be stated that the parameters describing vibration (max values a, A, rms) increase with the cutting speed v_f_. Moreover, for most cases, higher vibration values can be observed at m_a_ = 8 g/s (100%). The red circles mean "trend breakdown" in the form of stabilization or increase of a given vibration parameter. Such a situation takes place with a lower expenditure of the water-abrasive stream for m_a_ = 4 g/s.

### 3.2. Surface Roughness

**Figure 10:**
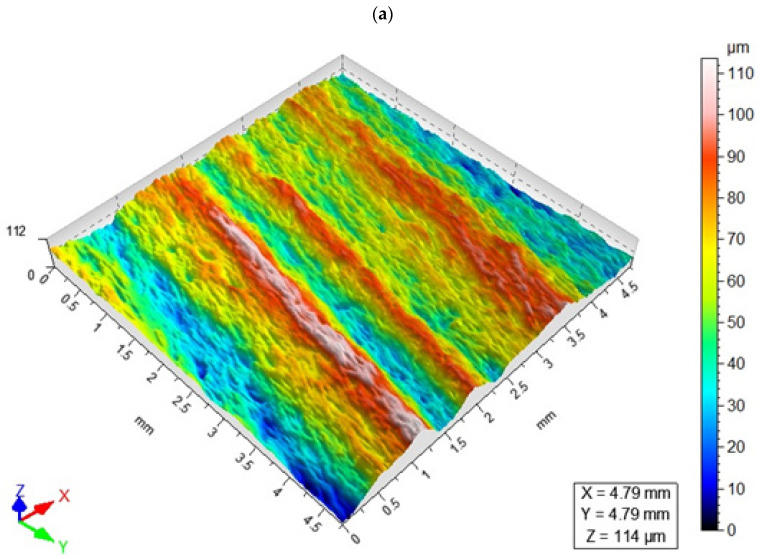

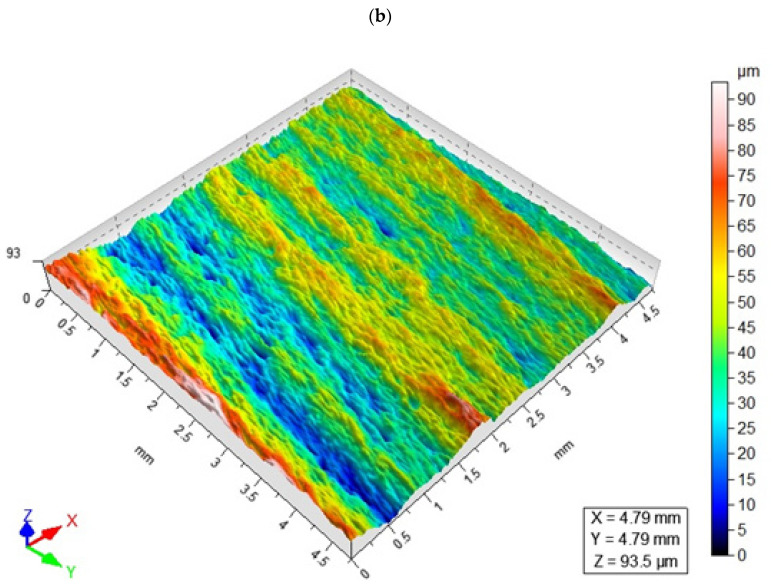
Surface topography maps for a constant cutting speed of v_f_ = 140 mm/min and different abrasive flow rates: (**a**) m_a_ = 4 g/s (50%), (**b**) m_a_ = 8 g/s (100%).

Figure 10 shows selected examples of the 3D surface topography of the AZ91D magnesium alloy specimens after AWJ cutting conducted with a constant cutting speed of v_f_ = 140 mm/min and variable abrasive flow rate.

The above surface topographies demonstrate that the use of an abrasive flow rate of 4 g/s (which is 50% of the maximum obtainable flow rate) results in higher elevations and depressions on the machined surface compared to a higher abrasive flow rate. The machining marks became even more uniform when the process was conducted with m_a_ = 8 g/s (which is 100% of the abrasive output).

**Figure 11:**
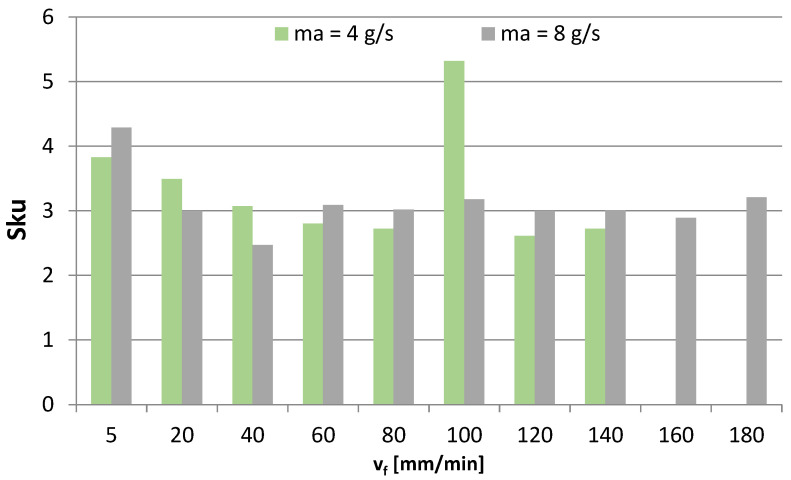
Cutting speed v_f_ and abrasive flow rate ma versus 3D roughness parameter Sku.

**Figure 12:**
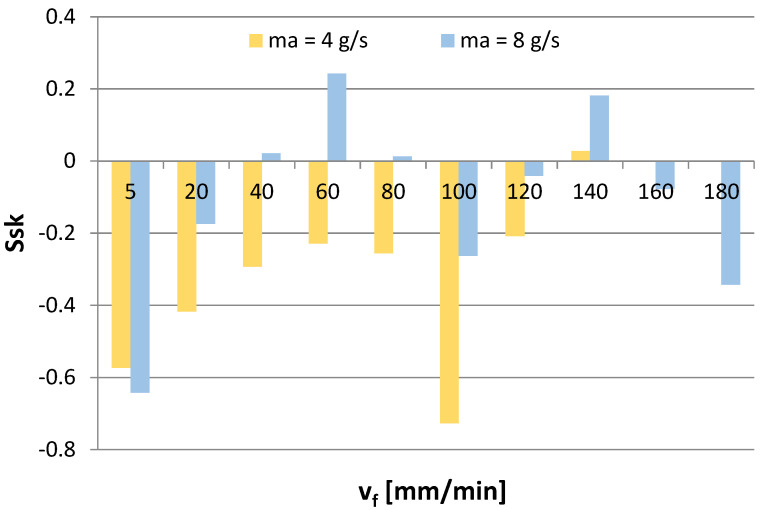
Cutting speed v_f_ and abrasive flow rate ma versus 3D roughness parameter Ssk.

**Figure 13:**
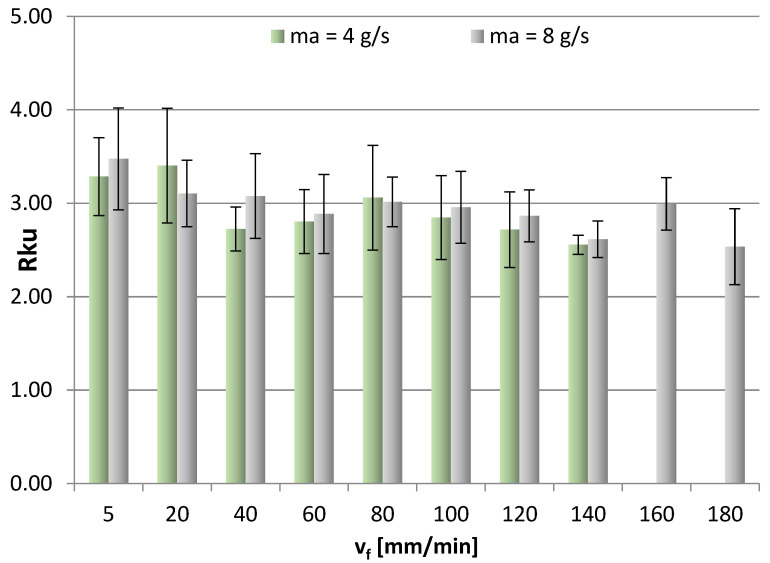
Cutting speed v_f_ and abrasive flow rate ma versus 2D roughness parameter Rku.

**Figure 14:**
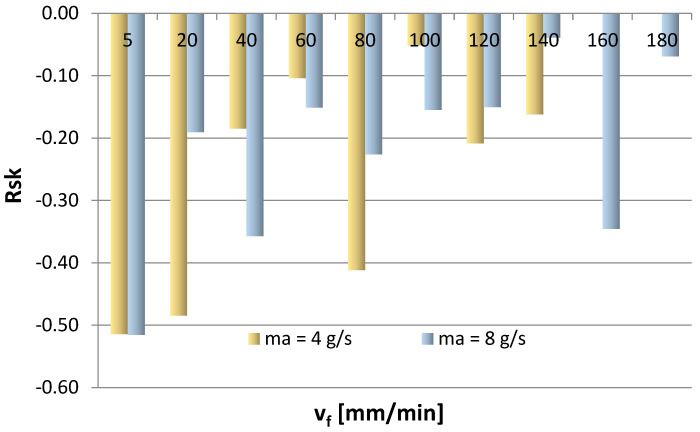
Cutting speed v_f_ and abrasive flow rate ma versus 2D roughness parameter Rsk.

Figure 11, Figure 12, Figure 13 and Figure 14 show the surface roughness results for 2D parameters (Rku, Rsk) and 3D parameters (Sku, Ssk). However, there is a lack of surface roughness data for the AWJM process conducted with a cutting speed of 160 mm/min and 180 mm/min and with an abrasive material flow rate of 4 g/s. This results from the fact that the samples were not cut with these speeds, which made it impossible to analyze the surface roughness of these samples.

Surface roughness parameters relating to profile height are important due to mating the surfaces of two machine components. From the point of view of their interaction, it is favorable that Sku takes positive values (Sku > 3 and Sku = 3), which is most clearly seen when v_f_ is 5 mm/min and 100 mm/min. Therefore, it can be concluded that for the analyzed case, the obtained surface is characterized by a low coefficient of friction. Less homogeneous results of the Sku kurtosis were obtained when m_a_ = 4 g/s.

However, when Ssk takes negative values, the friction becomes more intense, so, in this case, the favorable machining conditions should be m_a_ = 8 g/s and v_f_ =60 mm/min and 140 mm/min. Moreover, it is difficult to establish a clear trend for the Ssk parameter, as even though some results take positive values, most are negative values, which may indicate a plateau-like nature of the hills.

From the point of view of interaction (lower value of the friction coefficient) and proper operation of the mating components, it is favorable that the Rku parameter takes positive values. In an example given in Figure 11, for most cases, the kurtosis values above 3 indicate sharper vertices, which leads to a reduced friction coefficient of the mating surfaces (which is typical of surfaces with many sharp vertices). A positive skewness value means that the coefficient of friction is lower, whereas a negative skewness value (Rsk < 0) indicates that the friction is more intense, as can be observed in Figure 14. Moreover, it is difficult to establish a clear trend for the Rsk parameter based on its average values for a given abrasive output. Negative values of the Rsk parameter indicate a plateau-like nature of the hills.

### 3.3. Deflection Angle of Abrasive Water Jet

**Figure 15:**
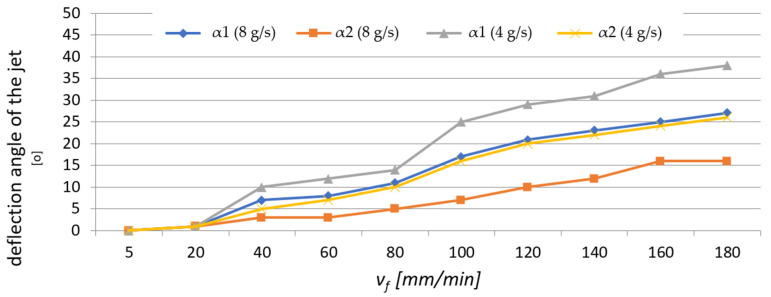
Abrasive water jet deflection angles α_1_ and α_2_ for different values of v_f_ and m_a_.

Figure 15 shows the abrasive water-jet deflection angle in the AWJ cutting of the AZ91D alloy. The results are shown for varying abrasive water-jet output and illustrate the jet deflection angles in two characteristic areas of the sample (angles α_1_ and α_2_).

The uneven distribution of kinetic energy results in the formation of visible machining marks on the bottom surface of the sample. The jet deflection angle is strongly correlated with the technological parameters of AWJM, as shown in Table 2.

An analysis of the data in Figure 15 demonstrates that as the abrasive flow rate increases, the α_1_ angle in the lower part of the sample surface (in the exit zone of the abrasive water jet) decreases. A similar trend can be observed for the α_2_ angle. This is probably related to a smaller amount of the abrasive agent as well as an overall lower jet intensity, and thus, its weaker impact on the cut surface of the sample. For the highest cutting speed, these differences were 11° for α_1_ and 10° for α_2_, respectively. These values are very close. However, when comparing the values of the angles for the same abrasive flow rate, it can be observed that those obtained for the jet deflection angle α_1_ were higher, regardless of the v_f_ value.

### 3.4. Numerical Modelling of Surface Roughness Parameters by Artificial Neural Networks

**Figure 16:**
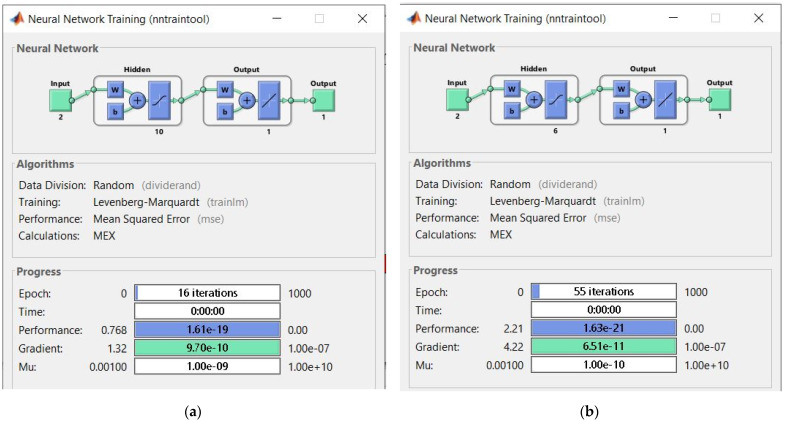
Neural network training structure and its parameters for analyzed roughness parameters: (**a**) Rku; (**b**) Sku.

**Figure 17:**
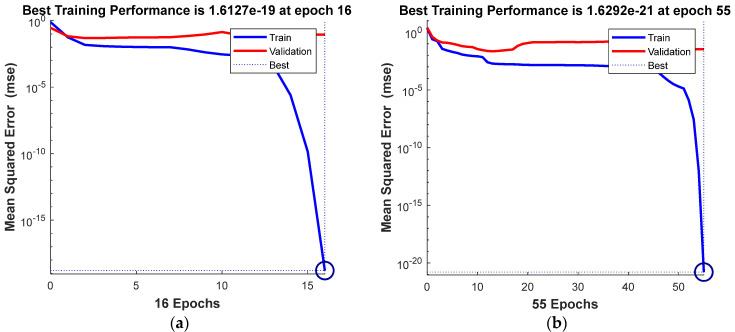
Best training performance of a predictive model for analyzed roughness parameters: (**a**) Rku; (**b**) Sku.

The best modeling results for the 2D parameter Rku were obtained with a network of 10 neurons, while for the 3D parameter Sku, it was the network containing 6 neurons in the hidden layer. The training structure of the neural networks and their parameters are shown in Figure 16 (Rku and Sku). Regarding the Rku parameter modeling, the best training performance (1.6127 × 10^−19^) was obtained with 16 iterations, which can be observed in Figure 17, while for the Sku parameter, it was achieved at 1.6292 × 10^−21^ with 55 iterations.

The quality of the obtained models was evaluated based on the regression value R as well as the mean squared error (MSE) and root mean square error (RMSE). Obtained values of the above parameters for the selected best models are listed in Table 3.

**Figure 18:**
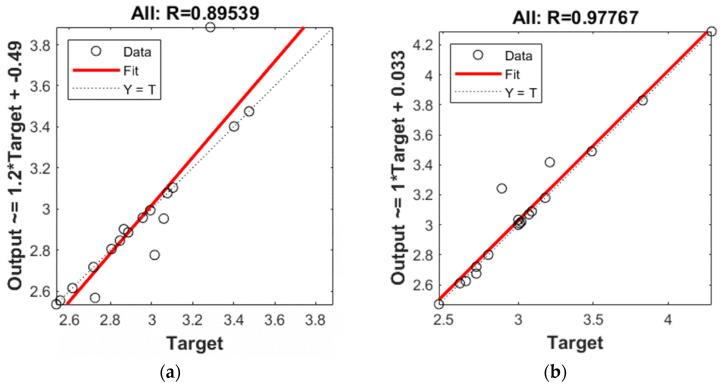
Correlation statistics for analyzed roughness parameters: (**a**) Rku; (**b**) Sku.

Correlation statistics for the experimental data and modeling results of the Rku roughness parameter are given in Figure 18. The overall correlation was R = 0.89539, which represents the degree of overlap between the measurement points and the fitting line with an ideal prediction line Y = T. Regarding the model prediction of the roughness parameter Sku, the overall correlation was R = 0.97767 (Figure 18). The use of modeling made it possible to predict values of the 2D (Rku) and 3D (Sku) roughness parameters.

The purpose of this part of the study was to model and predict selected 2D (Rku) and 3D (Sku) roughness parameters of the AZ91D magnesium alloy after AWJ cutting using artificial neural networks. The modeling results and the obtained regression values of R, MSE and RMSE demonstrate that the obtained networks have a satisfactory ability to predict these parameters.

## 4. Conclusions

The results of this study investigating the use of ANN modeling to predict the vibration and surface roughness of the AZ91D magnesium alloy after water-jet cutting led to the following conclusions:

- Greater skewness (jet deflection) was obtained at a lower abrasive flow rate of m_a_ = 4 g/s (50%), while higher values of the α_1_ angle (in the stream exit area) were also obtained for an abrasive flow rate of m_a_ = 4 g/s (50%), which can be explained by a weaker impact of the abrasive water jet on the machined surface.
- In the range of 60–140 mm/min, higher average values of the Sku roughness parameter were obtained at m_a_ = 8 g/s (100%), which means that this range of technological parameters should be applied to obtain low values of the friction coefficient.
- It is difficult to establish a clear trend for the Ssk parameter—although some results take positive values, most are negative, which may indicate a plateau-like nature of the hills.
- For most cases, higher average values of the Rku roughness parameter were obtained for the surfaces machined with a higher abrasive flow rate of m_a_= 8 g/s (100%) in the v_f_ range of 40–140 mm/min, which means that this range of technological parameters should be applied to reduce the coefficient of friction.
- The Rku kurtosis values exceeding and around 3 indicate sharper vertices, which reduces the coefficient of friction for the mating surfaces.
- It is difficult to establish a clear trend for the Rsk parameter (analysis of average values) for a given abrasive output; however, negative values of the Rsk parameter indicate a plateau-like nature of the hills.
- Regarding the range of vibration, it can be assumed, with simplification, that the parameters describing vibration (a, Aa, rms) increase with cutting speed v_f_.
- For most cases, higher vibration values were observed at m_a_= 8 g/s (100%), which can be explained by a greater impact of the abrasive water jet and a greater intensity of the cutting process.
- The input parameters for the modeling and prediction of selected 2D (Rku) and 3D (Sku) roughness parameters using artificial neural networks were variable technological parameters, i.e., the cutting speed v_f_ and the mass flow rate m_a_.
- Regarding the Rku parameter, the best parameters were obtained with the network with 10 neurons in the hidden layer for which MSE was 0.0252 and R = 0.89539; as for the 3D roughness parameter Sku, the best parameters were obtained with the network with 6 neurons in the hidden layer for which MSE was 0.0095 and R = 0.97767.
- The trained networks show a satisfactory ability to effectively model 2D and 3D surface roughness parameters of the AZ91D magnesium alloy.

The authors plan to continue research on AWJM for aluminum alloys in terms of other variable technological parameters and their impact on surface quality, as well as to develop relevant prediction models.

## Figures and Tables

**Table 1:** Technological parameters of the tests.

**Constant Technological Parameters**	
Abrasive	Garnet 80 mesh
Nozzle length	100 mm
Nozzle width	60 mm
Stand-off distance	3 mm
Pressure	350 MPa
**Variable Technological Parameters**	
Cutting speed v_f_	5, 20, 40, 60, 80, 100, 120, 140, 160, 180 mm/min
Abrasive flow rate m_a_	4 and 8 g/s

**Table 2:** AWJM parameters applied in the study.

Sample Number	v_f_ (mm/min)	m_a_ (g/s)	α_1_	α_2_
1	5	4	0	0
2	20		1	1
3	40		7	3
4	60		8	3
5	80		11	5
6	100		17	7
7	120		21	10
8	140		23	12
9	160		25	16
10	180		27	16
11	5	8	0	0
12	20		1	1
13	40		10	5
14	60		12	7
15	80		14	10
16	100		25	16
17	120		29	20
18	140		31	22
19	160		36	24
20	180		38	26

**Table 3:** R, MSE and RMSE regression values for modeled networks.

Model Number	Roughness Parameter	MSE	RMSE	R Training Data Set	R Validation Data Set	R All Data Set
1	Rku	0.0252	0.1586	0.99999	0.88494	0.89539
2	Sku	0.0095	0.0975	0.99999	0.90932	0.97767

## Data Availability

Not applicable.
